# Loss of BRUCE reduces cellular energy level and induces autophagy by driving activation of the AMPK-ULK1 autophagic initiating axis

**DOI:** 10.1371/journal.pone.0216553

**Published:** 2019-05-15

**Authors:** Lixiao Che, Xingyuan Yang, Chunmin Ge, Salim S. El-Amouri, Qi-En Wang, Dao Pan, Thomas J. Herzog, Chunying Du

**Affiliations:** 1 Department of Cancer and Cell Biology, University of Cincinnati College of Medicine, Cincinnati, Ohio, United States of America; 2 Division of Experimental Hematology and Cancer Biology, Cincinnati Children’s Hospital Medical Center, Cincinnati, Ohio, United States of America; 3 Department of Radiology, Ohio State University, Columbus, Ohio, United States of America; 4 Department of Pediatrics, University of Cincinnati College of Medicine, Cincinnati, Ohio, United States of America; 5 Division of Obstetrics and Gynecology, University of Cincinnati, Cincinnati, Ohio, United States of America; 6 University of Cincinnati Cancer Institute, Cincinnati, Ohio, United States of America; Univerzitet u Beogradu, SERBIA

## Abstract

Autophagy is an intracellular catabolic system. It delivers cellular components to lysosomes for degradation and supplies nutrients that promote cell survival under stress conditions. Although much is known regarding starvation-induced autophagy, the regulation of autophagy by cellular energy level is less clear. BRUCE is an ubiquitin conjugase and ligase with multi-functionality. It has been reported that depletion of BRUCE inhibits starvation-induced autophagy by blockage of the fusion step. Herein we report a new function for BRUCE in the dual regulation of autophagy and cellular energy. Depletion of BRUCE alone (without starvation) in human osteosarcoma U2OS cells elevated autophagic activity as indicted by the increased LC3B-II protein and its autophagic puncta as well as further increase of both by chloroquine treatment. Such elevation results from enhanced induction of autophagy since the numbers of both autophagosomes and autolysosomes were increased, and recruitment of ATG16L onto the initiating membrane structure phagophores was increased. This concept is further supported by elevated lysosomal enzyme activities. In contrast to starvation-induced autophagy, BRUCE depletion did not block fusion of autophagosomes with lysosomes as indicated by increased lysosomal cleavage of the GFP-LC3 fusion protein. Mechanistically, BRUCE depletion lowered the cellular energy level as indicated by both a higher ratio of AMP/ATP and the subsequent activation of the cellular energy sensor AMPK (pThr-172). The lower energy status co-occurred with AMPK-specific phosphorylation and activation of the autophagy initiating kinase ULK1 (pSer-555). Interestingly, the higher autophagic activity by BRUCE depletion is coupled with enhanced cisplatin resistance in human ovarian cancer PEO4 cells. Taken together, BRUCE depletion promotes induction of autophagy by lowering cellular energy and activating the AMPK-ULK1-autophagy axis, which could contribute to ovarian cancer chemo-resistance. This study establishes a BRUCE-AMPK-ULK1 axis in the regulation of energy metabolism and autophagy, as well as provides insights into cancer chemo-resistance.

## Introduction

The BIR repeat containing ubiquitin-conjugating enzyme (BRUCE) is a high molecular mass protein (528 kDa) with multiple cellular functions. BRUCE was initially identified as a member of the inhibitor of apoptosis protein (IAP) family owing to having a revolutionarily conserved anti-apoptotic BIR domain, which is present in all IAP family members [1, 2]. In the IAP family, BRUCE is unique because it is the only member that also contains a ubiquitin conjugating (UBC) domain near its C-terminus, which makes BRUCE with both ubiquitin (Ub) conjugating (E2) and ligase (E3) activities [1]. BRUCE catalyzes the ubiquitination of proteins for the regulation of apoptotic activity [3–8]. As an IAP, BRUCE overexpression inhibits apoptosis by its binding and thereby inhibiting processed/activated caspases-3, 7, and 9 activities, the major executioners of apoptosis [6, 9, 10]. BRUCE suppresses apoptosis also by ubiquitinating and promoting proteasomal degradation of pro-apoptotic caspase-9 and Smac/Diablo (IAP antagonist) [11–13] [6, 9]. Studies of *Bruce* in global *Bruce* knockout (KO) mice revealed both functions of *Bruce* in anti-apoptosis and maintaining proliferation. The *Bruce* KO mice are embryonic lethal and die on E16.5–17.5. Prior to their death, the viable homozygous KO embryos and the extraembryonic tissues of placenta and yolk sac exhibit increased levels of apoptosis and reduced amount of cell proliferation [3, 4, 9, 14]. The anti-apoptotic function of BRUCE is evolutionarily conserved in mammals and *Drosophila*, as its Drosophila homolog *dBruce* suppresses cell death induced by Reaper and Grim, which are the functional homologues of Smac/DIABLO [15, 16].

Despite the challenges with characterization of high molecular mass proteins, studies have continued to uncover several non-IAP functions for BRUCE. During cytokinesis, BRUCE promotes the final stage of cytokinesis, the abscission [17]. Being localized to the midbody, BRUCE forms a platform to interact with mitotic regulators and components of the vesicle-targeting machinery to assist their delivery to the site of abscission [17]. In addition to cytokinesis, our group has reported another critical non-IAP function for BRUCE in maintaining genome stability. In this role, BRUCE is required for activation of the ATM-DNA damage response in response to ionizing radiation (IR) induced DNA double-strand breaks (DSBs) [18, 19]. This DNA damage response function of BRUCE is separate from its function in anti-apoptosis, because it does not require the anti-apoptotic BIR domain [18, 19]. The connection between BRUCE and ATM-DNA DSB activation signaling occurs via recruitment of BRIT1/MCPH1 to site of DNA DSBs [18, 19]. BRIT1 is a tumor suppressor and promotes DNA damage response [20, 21]. BRUCE acts as a nuclear scaffold for the assembly of a tri-molecular complex consisting of BRUCE-USP8-BRIT1. Following IR induction, both the scaffolding and the UBC activities of BRUCE promote USP8-mediated deubiquitination of BRIT1, the subsequent translocation of deubiquitinated BRIT1 to sites of DNA damage and BRIT1-dependent recruitment of SWI-SNF chromatin remodeler to the damaged sites resulting in chromatin relaxation that activates the ATM pathway and homologous recombination (HR). As a result, BRUCE deficient cells exhibit genome instability with a significantly higher incidence of abnormal chromosomal gaps, breaks, polyploidy and telomere end-end association [18, 19]. Also due to the HR deficiency, BRUCE deficient cells are sensitive to DNA damaging chemotherapeutic agents in breast cancer cells [22]. BRUCE maintains genome stability also through a BRUCE-ATR signaling axis to facilitate replication stress response [23]. BRUCE, recruited to induced DNA damage sites, facilitates ATR-dependent signaling events during replication stress, including activation of ATR itself, phosphorylation of its downstream targets CHK1 and RPA, and the mono-ubiquitination of FANCD2. BRUCE deficiency resulted in stalled DNA replication forks and increased firing of new replication origins [23]. The impact of the BRUCE-ATR-DDR signaling axis is crucial in vivo for suppression of hepatocellular carcinoma. Liver-specific knockout of murine *Bruce* impaired ATR activation in the liver and exacerbated hepatic inflammation, fibrosis and hepatocellular carcinoma, initiated by hepatocarcinogen and genotoxin Diethylnitrosamine (DEN) [23].

There is a complex but close relationship among apoptosis, DNA damage/genome instability, cellular energy levels and autophagy. Autophagy is an evolutionarily conserved intracellular catabolic pathway in eukaryotes for clearance of damaged or superfluous intracellular components into basic biomolecules under normal and stress conditions [24, 25]. Autophagy can be sub-classified as basal or induced autophagy [26–28]. Basal autophagy occurs constitutively at low levels under normal growth conditions, and is critical for intracellular quality control through the constitutive turnover of cytoplasmic components. In contrast to basal autophagy, induced autophagy occurs via starvation or glucose and amino acid deprivation among other inducers, and it plays a pleotropic role, including pro-survival during adaptation to unfavorable growth conditions, development, differentiation, immune homeostasis, and defense against pathogens [26, 27]. Both basal and induced autophagy are co-occurring cellular processes of production and degradation of autophagosomes. Therefore, basal or induced autophagic activity cannot be simply deduced by changes in autophagosomal numbers or the autophagic marker LC3-II protein level (lipidated; present on autophagosomes), but rather require measurement of autophagic flux, which is a comparison of differential numbers of autophagosomes before and after the addition of lysosome blockers such as chloroquine (CQ) or bafilomycin, both of which inhibit lysosomal enzyme activities and thereby prevent degradation of cargoes in autolysosomes [29–32].

The ULK1 (unc-51-like kinase 1) protein complex drives the formation of the initial membrane structure called phagophores. The phagophores further develop to produce the landmark structure of double-membraned autophagosomes. Autophagosomes capture cargos of organelles, lipids, carbohydrates and proteins, and by fusion with the lysosome and form autolysosomes, these cargos inside autophagosomes are degraded by lysosomal enzymes. The degraded products of intracellular cargos are returned to the cytoplasm and merge into multiple metabolic pathways, thereby completing the autophagic process [24, 25]. These degraded products are hypothesized to be the energy source or building blocks for cell survival upon starvation or other stress conditions, thereby maintaining energy balance and tissue homeostasis [24, 25].

The AMP-activated protein kinase (AMPK) is a critical cellular energy sensor. AMPK is activated by a reduced cellular energy state. Increased levels of ADP and AMP are signaling indicators of a compromised cellular energy state and that ADP and AMP cause allosteric activation of AMPK [33–36]. When cellular energy is low or depleted, AMPK directly phosphorylates the autophagy-initiating kinase ULK1 and thereby activates the catabolic autophagy for production of cellular energy to maintain energy homeostasis [37].

BRUCE also impacts autophagy. Studies of the autophagy function of *dBruce* in *Drosophila Melanogaster* indicated that *dBruce* inhibits autophagy under nutrient-rich conditions. However, during oogenesis when autophagy activity is needed, the inhibition of *dBruce* on autophagy is revoked and consequently increases autophagy activity. This activation of autophagy by loss of *dBruce* function was verified by autophagic flux assays with treatment of the pharmacological inhibitors of autophagy 3-MA (3-methyladenine; inhibitor of class III PI3K required for activation of autophagy) and Bafilomycin A1 (inhibitor of lysosome function) [38]. In contrast to the autophagy inhibitory function of *dBruce*, a recent study of mammalian BRUCE showed that BRUCE promotes starvation-induced autophagy in mouse embryonic fibroblasts (MEFs) by participating in the fusion step of autophagosomes with lysosomes [39].

However, the impact of BRUCE on autophagy in mammalian cells under normal culture condition (without starvation) remains unclear. It is believed that autophagy could have distinct cellular functions under different culture conditions, but this distinction remains undefined [27]. Therefore, we sought to investigate the impact of BRUCE on autophagic activity in complete medium, and delineate the underlying mechanisms, while elucidating the implications of autophagy in cancer cell survival.

## Materials and methods

### Cell line generation and cell culture

U2OS-shBRUCE-GFP-LC3 is a stable cell line derived by transfection of the previously published U2OS-shBRUCE cell line (DOX-inducible expression of shBRUCE U2OS cells, [18, 19]) with a GFP-LC3 expression construct by Lipofectamine 2000 (Thermo Fisher Scientific). GFP-positive stable cells were sorted by flow cytometer and verified by immunoblot and immunofluorescence staining. U2OS-shBRUCE cell line with reconstituted expression of siBRUCE-resistant FLAG-tagged BRUCE was reported previously [18, 19]. Human liver cell line THLE2, ovarian cancer cell lines PEO1 and PEO4 were purchased from ATCC. mCherry-GFP-LC3 MEFs were gift from Dr. Xue-Jun Jiang at The Sloan Kettering Cancer Institute.

U2OS-shBRUCE and its derived cell lines are cultured in DMEM high glucose medium with 10% FBS (tetracycline-free) and 1% penicillin/streptomycin. The rest cell lines are cultured in DMEM high glucose medium with 10% FBS (non-tetracycline-free) and 1% penicillin/streptomycin. All cell lines are kept at 37°C in a CO2 (5%; vol/vol) incubator.

### BRUCE knockdown in U2OS-shBRUCE-GFP-LC3, THLE2, mCherry-GFP-LC3 MEFs and PEO4

To induce BRUCE knockdown in U2OS-shBRUCE-GFP-LC3 cells, DOX (1μg/ml) was added for 4 days. U2OS cells without DOX treatment were used as the control. Two sequence independent siRNAs targeting BRUCE expression were used on U2OS cells for verification purposes (siBRUCE#2: GACCUUAAUGGAAUCUUGUdTdT, siBRUCE#3: GUUAUGAGCUGCUUGUAGAdTdT). BRUCE depletion in THLE2 cells was performed by lentivirus mediated shRNA targeting BRUCE, as described previously [22]. For BRUCE knockdown in mCherry-GFP-LC3 MEFs and PEO4 cells, a siRNA corresponding to the BRUCE sequence (siBRUCE: GGCACAGCAGCUCUUAUCAdTdT) was used. The control siRNA sequence is UUCUCCGAACGUGUCACGUdTdT. Transfection was performed with Lipofectamine RNAiMAX Regeant (Thermo Fisher Scientific).

### Western blot analysis

Cells were placed on ice, washed twice with cold PBS and lysed with NETN buffer [50 mM Tris-HCI (pH 8.0), 150 mM NaCl, 1 mM EDTA, 0.5% NP-40] plus protease inhibitors. 30–50 μg protein of each sample was used for analysis by immunoblotting. Quantification of western blot films was performed with Image J, with the measure of controls set as 1. Antibodies used include: BRUCE (Bethyl, A300-367A), LC3B (Cell signaling, 2775 and 3868), phospho-ULK1 (Cell signaling, 5869), phospho-AMPKα (Cell signaling, 2531), ULK1 (Cell signaling, 8054), AMPKα (Cell signaling, 5832), GFP (Santa Cruz, sc-9996), FLAG (Sigma, A8592), GAPDH (Cell signaling, 5174), β-Actin (Cell signaling, 3700), α-Tubulin (Sigma, T9026).

### Immunofluorescence analysis

Cells were fixed with cold methanol and then incubated with primary antibodies overnight and stained with a secondary antibody (conjugated with Alexa Fluor 488). The following antibodies were used: ATG16L (Cell signaling, 8089), LC3B (Cell signaling, 2775 and 3868). Images were collected using a confocal microscope (Zeiss LSM 710).

### Transmission electron microscopy (TEM) analysis

U2OS-shBRUCE cells, left untreated or treated with DOX to induce BRUCE knockdown, were subject to TEM analysis. TEM sample preparation follows a widely used protocol. Specifically, one million cells were pelleted and fixed for 2 hr. in 2% paraformaldehyde (TEM grade) and 2.5% glutaraldehyde (TEM grade). After washing for 2 x 5 mins in PBS, pellet cells were exchanged of chemical until being embedded in 2% low temperature agarose. After dehydration in graded ethanol from 70% to 100%, samples were embedded in resin, and sections were cut at 70 nm on an ultramicrotome and examined using a JEOL JEM 1230 TEM. Images were taken with an AMT Advantage Plus 2K x 2K digital camera.

### Cell survival assay

PEO1 and PEO4 cells were seeded in 96-well plates at an initial density of 5,000 cells/well. On the following day, the cells were treated with increasing doses of cisplatin for 48 hrs. Then the cells were washed with PBS, fixed with 3.7% formaldehyde for 30 min and stained with 1% methylene blue for 30 min. The plates were rinsed in running water, and then left to dry. 100 μl solvent (10% acetic acid, 50% methanol and 40% water) was added to each well, and the optical density of the released color was read at 660 nm. The relative cell survival was conducted in three independent repeats and calculated with the values of mock-treated cells set as 100%.

### Measurement of AMP/ATP ratio

U2OS-shBRUCE-GFP-LC3 cells were treated with or without DOX. Four days later, cells were collected and assayed using the ATP/ADP/AMP Assay Kit (Biomedical Research Service Center, University at Buffalo, Buffalo, NY).

### Fluorometric enzyme assay for α-L-iduronidase and β-hexosaminidase

The catalytic activity of *α-L-iduronidase* (IDUA) or β-hexosaminidase (Hex) was determined with a fluorometric enzyme assay as previously described in [40] and [41], respectively, with modifications. For IDUA assay, cell pellets were homogenized in lysis buffer (150 mM NaCl and 50 mM Tris-HCl with 1% Triton X-100) using an Ultrasonic Processor (GE). Aliquots of cleared lysate were incubated at 37°C for 60 minutes with 2.5 mM fluorogenic substrate, 4-methylumbelliferyl (4MU) α-L-idopyranosiduronic acid sodium salt (Toronto Research Chemicals) in 0.4 M NaFormate buffer, followed by the addition of glycine carbonate buffer (0.1 M, pH 10.5) to stop the reaction. For determination of β-hexosaminidase activity, cells were homogenized in 0.9% sodium chloride solution containing 1% Triton X-100, followed by dilution in distilled water. The diluted homogenates were incubated with 1.2 mM 4MU-β-N-acetylglucosaminide (Sigma) in 10 mM citrate/20mM phosphate buffer for 1 hr. at 37°C. The reaction was stopped by the addition of glycine carbonate buffer. The fluorescent product released from each reaction was analyzed with an emission wavelength of 450 nm and an excitation wavelength of 365 nm using SpectraMax M5 fluorometer (MDS Analytical Technologies). Samples were assayed in duplicate or triplicate reactions, together with buffer controls in parallel. Protein concentration was determined by Pierce BCA Protein Assay (Thermo Fisher Scientific). One unit of enzyme activity was defined as the release of 1 nmol of 4MU in a 1-hr reaction at 37°C. Intracellular specific enzyme activity was calculated as unit/mg protein.

### Data analysis

The results are expressed as the means ± standard deviation (SD) of the determinations. The statistical significance of the difference was determined by Student’s *t*-test (two-tailed).

## Results

### Depletion of BRUCE expression increases autophagic activity indicated by the LC3 turnover and GFP-LC3 cleavage assays

**Fig 1:**
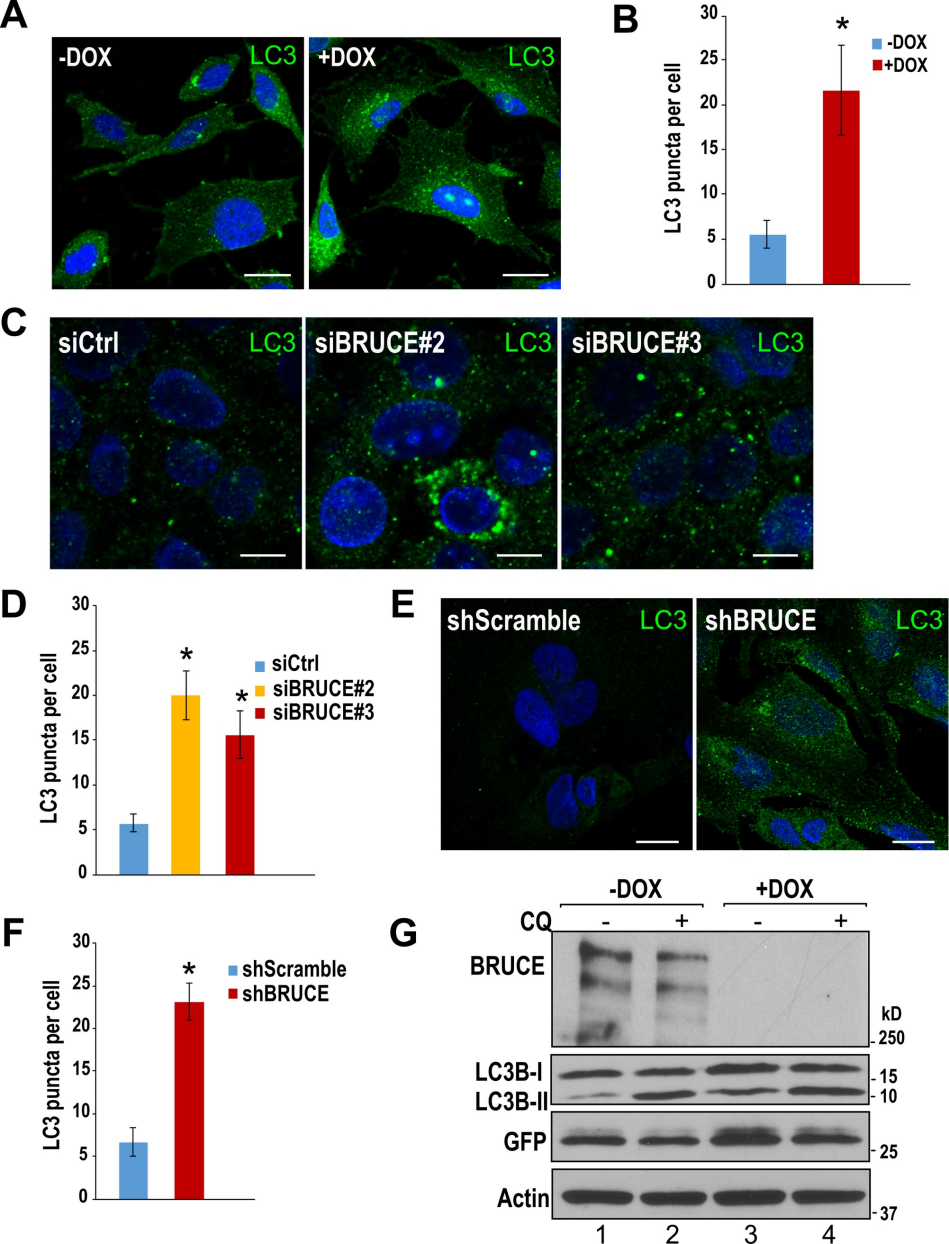
LC3 turnover assay showing an elevated autophagy activity induced by BRUCE depletion. (A) U2OS-shBRUCE-GFP-LC3 cells (clone #16, maintained in complete medium) were left untreated or treated with DOX to induce knockdown of BRUCE expression, followed by immunostaining with a LC3B antibody (green) and counter stained with DAPI (blue), Bar: 20 μm. (B) Semi-quantification of LC3B puncta numbers (immunofluorescent puncta) in each cell. *, p<0.05. (C) U2OS-shBRUCE-GFP-LC3 cells were transfected with control (siCtrl) and two distinct siRNAs targeting BRUCE (siBRUCE#2 and siBRUCE#3). LC3B puncta were immunostained and the results were quantified in (D). *, p<0.05. Bar: 10 μm. (E) BRUCE knockdown was performed with lentivirus mediated shRNAs (shScramble and shBRUCE) in starved human liver cell line THLE2. LC3B puncta were immunostained and the results were quantified (F). *, p<0.05. Bar: 20 μm. (G) DOX treated U2OS-shBRUCE-GFP-LC3 cells were incubated with CQ (50 μM, 2 hr) to block lysosome fusion and the total cell lysates were immunoblotted with indicated antibodies.

During the formation of autophagosomes, the LC3 protein is conjugated with phospholipid and the resulting lipidated LC3B-II protein is localized on autophagosomes and autolysosomes. To prepare cells for autophagy analysis, we used our previously published U2OS cell line that expresses a Doxycycline (DOX)-inducible expression of shBRUCE to induce BRUCE depletion [18, 19]. From this cell line, we derived a new cell line that stably expresses the GFP-LC3B vector (U2OS-shBRUCE-GFP-LC3B; see materials for details). To examine whether BRUCE impacts autophagy, cells maintained in complete medium were treated with DOX to induce BRUCE knockdown, and the number of LC3 puncta was monitored. We observed a significant increase in the number and intensity of LC3 puncta in BRUCE depleted cells compared to control (-DOX) (Fig 1A and 1B). To verify this result, we performed BRUCE knockdown with two independent siRNAs (siBRUCE#2 and siBRUCE#3) that their targeting sequences are different from shBRUCE. Comparable results were obtained with both siRNAs (Fig 1C and 1D). Similar results were also observed in different cell lines, including the normal human liver THLE2 cells (Fig 1E and 1F). It is known that an increase in the number of LC3 puncta can be a result of an increased autophagic activity or a blockage of the fusion of autophagosomes with lysosomes [29]. To distinguish between these two possibilities, we conducted an autophagic flux assay to monitor the LC3B turnover by immunoblotting analysis. CQ treatment of the cells induced an expected increase of the LC3B-II protein levels as compared to controls (Fig 1G, lane 2 vs 1), and these increased LC3B-II levels represent basal autophagic activity. Depletion of BRUCE expression resulted in an increase in the levels of LC3B-II as compared to the BRUCE-proficient control (Fig 1G, lane 3 vs lane 1), supporting the observed increase in autophagosome numbers as shown in Fig 1A and Fig 1B. Interestingly, in BRUCE depleted cells, CQ treatment resulted in a further increase in the LC3B-II protein levels (Fig 1G, lane 4 vs 3), implying an elevated autophagic flux, i.e., increased autophagic activity. Next we took the advantage of having the GFP-LC3 fusion protein expressed in the cells to verify the enhanced autophagic activity, and test whether a proficient fusion of autophagosomes with lysosomes is achieved by using the generation of free GFP protein as the indicator. Only when the fusion step is successful will free GFP be generated through cleavage of the GFP-LC3 fusion protein by lysosomal enzymes. As GFP is resistant to lysosomal degradation, it remains at full-length after being generated from GFP-LC3 fusion protein. Thus, free GFP can be used to discriminate between elevated autophagy activity which increases free GFP protein production, or blockage of the fusion which decreases protein production [42, 43]. Consequently, we conducted immunoblotting of the same membrane with a GFP antibody. The results demonstrated elevated free GFP protein levels in BRUCE depleted cells as compared to controls (Fig 1G, lane 3 vs 1). Increased free GFP protein was verified to be generated within lysosomes. When lysosomal function was inhibited by CQ, the amount of free GFP protein was reduced (Fig 1G, lane 4 vs 3). Together, these data indicate that BRUCE depletion promotes the increase of autophagy activity rather than blockage of the fusion step when cells are cultured in complete medium.

### Depletion of BRUCE increases the induction of autophagy

**Fig 2:**
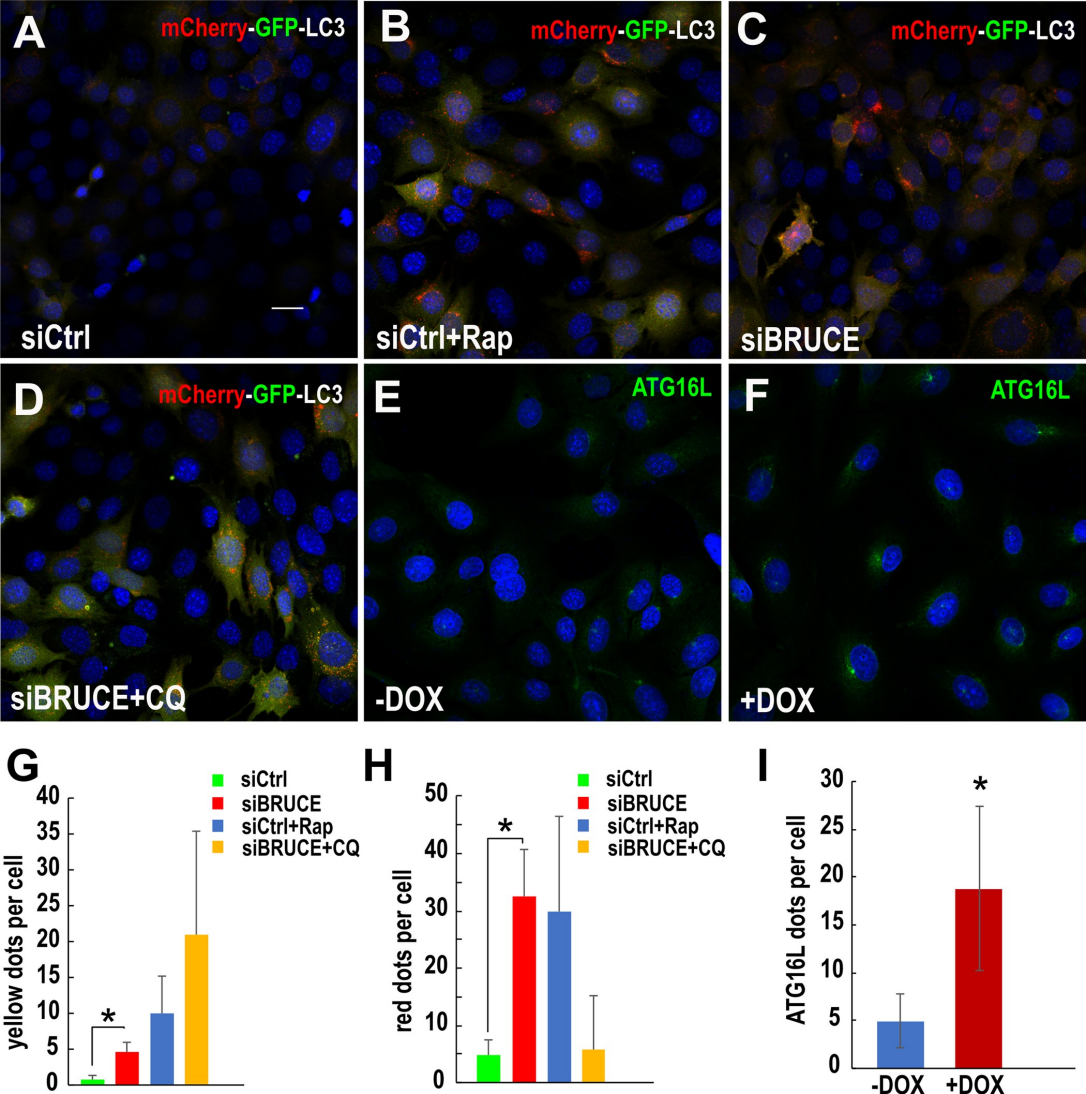
The mCherry-GFP-LC3 assay and elevated phagophore formation showing enhanced induction of autophagy by BRUCE depletion. Mouse embryonic fibroblasts (MEFs) expressing mCherry-GFP-LC3 were transfected with control siRNA (siCtrl) (A and B) or siBRUCE (C and D) followed by treatment with rapamycin (Rap) (B, 2 μM, 4 hr) or CQ (D, 200 μM, 2 hr). After cell nucleus staining with DAPI, cells were examined for autophagy flux utilizing confocal microscopy. Autophagosomes in the cytoplasm emit yellow fluorescence versus red when inside lysosomes. Rapamycin (removal of the inhibitory role of mTOR on autophagy) served as a positive control for enhanced autophagy function (i.e., increased both yellow and red fluorescence dots). (E and F) Endogenous ATG16L was immunostained using an anti-ATG16L antibody in U2OS-shBRUCE-GFP-LC3 cells with BRUCE expressed (-DOX) or depleted (+DOX). (G and H) Quantifications of autophagosomes (yellow dots) and autolysosomes (red dots) in Fig 2A–2D. *, p<0.05. (I) Quantification of ATG16L dots in Fig 2E and 2F. Bar: 20 μm. *, p<0.05.

To further investigate at which stage of the autophagic process that BRUCE depletion has caused increased activity, we employed the dual color mCherry-GFP-LC3 autophagy flux assay coupled with autophagy activator and inhibitor treatment [44]. In this assay, the autophagic flux can be traced by the color changes of the red (mCherry) and green (GFP) fluorescence. Although the GFP protein is resistant to lysosomal protease digestion and remains intact as a full-length GFP protein, the low pH inside the lysosome quenches the fluorescent signal of GFP [29]. In contrast, the red fluorescence of mCherry is stable in acidic compartments, and remains detectable inside the lysosome [29]. Therefore, those autophagosomes incorporated with mCherry-GFP-LC3 proteins display yellow fluorescence in the cytoplasm, whereas those delivered into the lysosome exhibit red fluorescence [29]. Based on these features, an elevated induction of autophagy is expected to result in an increase in the florescence of both yellow (autophagosomes) and red (autolysosomes), whereas blockage of the fusion step results in increased in yellow but reduction of red fluorescence [29]. BRUCE expression was depleted by siRNAs in MEFs which stably express the mCherry-GFP-LC3 reporter [44]. The fluorescence changes were monitored under different chemical treatment. Rapamycin is an activator of autophagy because it inhibits mTOR (mammalian target of rapamycin) activity, and mTOR inhibits autophagy induction [45]. Therefore, rapamycin treatment serves as a positive control for increased induction of autophagy. MEFs treated with rapamycin exhibited expected enhancement in the induction of autophagy by displaying elevated yellow and red fluorescence as compared to controls (Fig 2B vs Fig 2A). Interestingly, similar to the rapamycin treatment, BRUCE depletion also increased both the yellow and red fluorescence (Fig 2C), indicating that BRUCE depletion promotes the induction of autophagy. Further, the promotion of autophagy induction was validated by observation of increased autophagic flux following the CQ treatment, because when the fusion step is blocked by CQ, BRUCE-depleted cells displayed increased yellow fluorescence, i.e. accumulation of autophagosomes from elevated induction of autophagy (Fig 2D). These results were quantified in Fig 2G and 2H.

### Depletion of BRUCE promotes formation of the initiating autophagic phagophores

Induction of autophagy starts with the formation of initiating phagophores/isolation membranes, and the expansion of the phagophores through lipid acquisition to become autophagosomes [46]. Thus, phagophore formation is an essential process for autophagic induction and elevated autophagic activity. Central to phagophore formation is the ATG16L protein, which interacts with ATG12-ATG5 proteins to form an E3-like enzyme complex for the conjugation of phosphatidylethanolamine (PE) to LC3 to produce the membrane-bound, active form LC3-II. The ATG16 complex promotes elongation of the nascent autophagosomal membrane leading to autophagosome formation. Thus, recruitment of ATG16L with phagophore membrane serves as a specific phagophore marker [46–48]. To gain insight into the molecular basis underlying the elevated autophagic induction mediated by BRUCE depletion, we examined the impact of BRUCE on the recruitment of ATG16L to phagophores. Immunofluorescence staining showed elevated levels of ATG16L on the initiating phagophore structures in BRUCE depleted cells (Fig 2F) compared to controls (Fig 2E) with these results quantified (Fig 2I). Thus, BRUCE depletion promotes the formation of phagophores, thereby increasing the initiation of autophagosome formation and the subsequent enhancement of autophagic activity.

### Depletion of BRUCE elevates autophagic activity examined by electron microscopy

**Fig 3:**
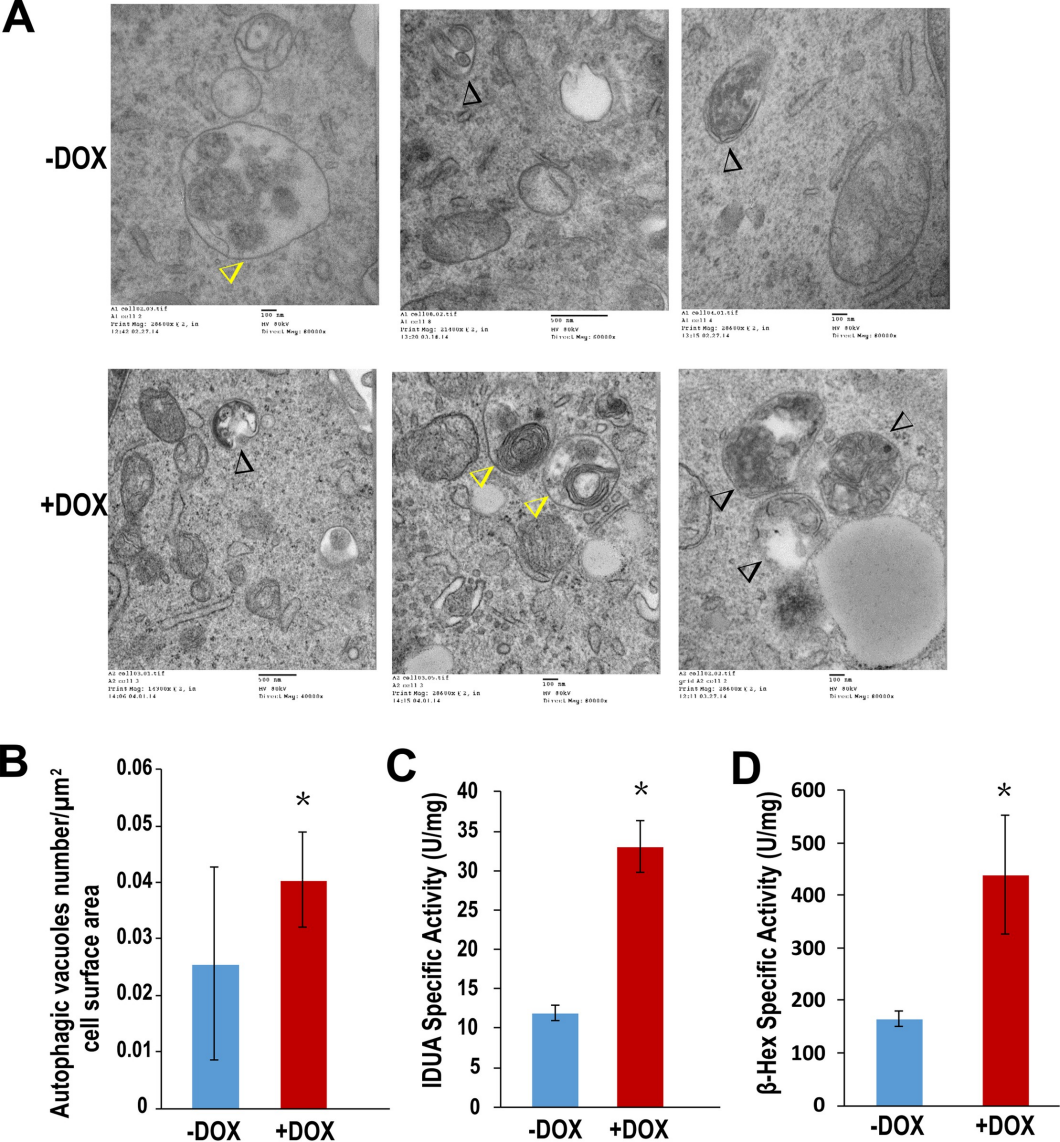
Transmission electron microscopic (TEM) analysis of autophagosomes. U2OS-shBRUCE cells with BRUCE depleted (+DOX) or proficient (-DOX) were processed for TEM analysis (A and B, see Materials for details) and analyzed for the number of autosomes and autolysosomes under TEM showing autophagosomes (black open triangles) and autolysosomes (yellow open triangles) with results quantified to cell areas per square micrometer. *, p<0.05. (C and D) Two lysosomal enzyme activities were measured in BRUCE depleted cells. *, p<0.05.

Transmission electron microscopy (TEM) is a classic method for detecting autophagic activity with high resolution that is superior to fluorescence microscopy. Furthermore, an advantage of microscopy is that there is no need for antibody staining, which avoids potential antibody cross reactivity with subcellular structures other than the autophagic compartments. Additionally, direct visualization of autophagosomes and their cargos enhances the reliability of autophagosome and autolysosome counting [49]. The ultrastructure of an autophagosome is a double-membrane vesicle that contains undigested cargo of cytoplasmic materials such as mitochondria and fragments of endoplasmic reticulum. In contrast, an autolysosome has single-membrane and contains cytoplasmic cargo at various stages of degradation [29]. Elevated induction of autophagy increases the numbers of both autophagosomes and autolysosomes, whereas inhibition of the downstream fusion step only leads to the increase of autophagosome numbers, and a decrease in autolysosome numbers [29]. Based on these features, TEM examination showed increased numbers of both autophagosomes and autolysosomes per microscopic field in BRUCE depleted U2OS cells as compared with control cells (Fig 3A). Considering the possibility that larger cells may proportionally have more autophagosomes than smaller cells within the same cell culture population, we quantified the numbers of autophagic vacuoles (autophagosome and autolysosome combined) per cell, divided that by the size of the cell and calculated the number of autophagic vacuoles per μm^2^. The comparison demonstrated a significant increase in the numbers of both autophagosomes and autolysosomes in BRUCE depleted cells as compared to controls (Fig 3B). These TEM results confirmed an elevated induction of autophagy provoked by BRUCE depletion.

### Depletion of BRUCE elevates lysosomal enzyme activities

Autophagosomes fuse with lysosomes, and the contents inside of autophagosomes are degraded by lysosomal enzymes. The elevated autophagic activity could demand a higher lysosomal degradative capability to degrade cargos. Lysosomes contain approximately 50 different degradative enzymes that hydrolyze lipids, polysaccharides, proteins and other macromolecules of the autophagic cargos. The intracellular degradative products are subsequently released to the cytosol for reutilization as energy or building blocks in metabolism. Therefore, to examine whether the lysosomal enzymatic activity is altered by BRUCE depletion, α-L-iduronidase (IDUA) was selected as one representative enzyme because it is a major lysosomal glycoprotein enzyme essential for the degradation of large sugar molecules, i.e., glycosaminoglycans (https://www.ncbi.nlm.nih.gov/gene/3425). Cells depleted of BRUCE displayed a 2.8-fold increase of the IDUA activity as compared to BRUCE proficient cells (Fig 3C). To further understand whether it is an overall increase of the lysosomal enzymatic activity or an exclusive elevation of the IDUA function, we examined a lysosomal lipase *β*-hexosaminidase (*β*-Hex), which is involved in the hydrolysis of lipids, the GM2 ganglioside (https://www.ncbi.nlm.nih.gov/gene/3074). The *β*-Hex activity also increased by 2.7-fold (Fig 3D). Together these results confirm an elevation of the lysosomal degradation capacity by BRUCE depletion.

### Depletion of BRUCE reduces cellular energy and stimulates the induction of autophagy mediated by the AMPK-ULK1 axis

**Fig 4:**
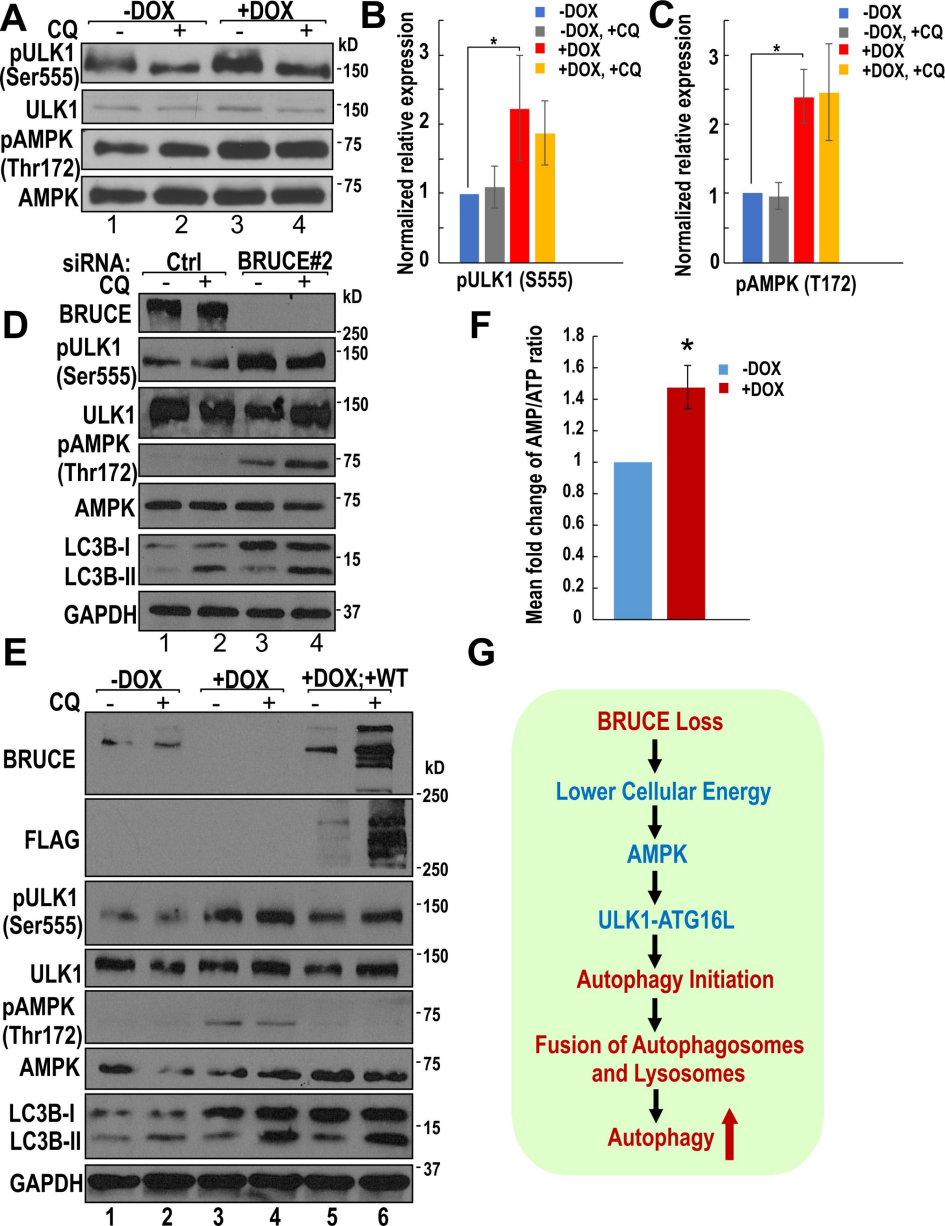
BRUCE depletion induces autophagy via the AMPK-ULK1 mediated activation of the autophagy axis. (A) U2OS-shBRUCE-GFP-LC3 cells were treated with or without DOX. CQ (50 μM, 2 hr) was then added to block lysosome fusion. Cell lysates were immunoblotted with antibodies as indicated. (B and C) Immunoblot results with multiple replicates were quantified for pULK1 (B) and pAMPK (C). *, p<0.05. (D) U2OS cells treated with siBRUCE #2 to knockdown BRUCE expression were immunoblotted with indicated antibodies. (E) Rescue experiments in U2OS-shBRUCE cells reconstituted with a siBRUCE-resistant FLAG-BRUCE (+DOX; +WT), with the cell lysates immunoblotted with indicated antibodies. (F) Cell lysates were assayed for the ratio of AMP/ATP and mean fold change was compared in control (-DOX) and BRUCE KD (+DOX) cells. Control was normalized as 1. Three independent experiments. *, p<0.05. (G) A working model showing a BRUCE-AMPK-ULK1 axis in the regulation of energy metabolism and autophagy.

BRUCE depletion promotes formation of the ATG16L-containing initiating phagophore structures (Fig 2F and 2I). The formation of phagophores can be activated by the conserved kinase complex containing the autophagy-initiating kinase ULK1. Specifically, in response to poor nutrient condition or ATP depletion, the ULK1 kinase is activated by phosphorylation at several primary sites (Ser555 and Ser777 as examples) by the energy sensor kinase AMPK [50–54]. Once activated, the ULK1 kinase phosphorylates the downstream autophagy proteins to promote formation of the ATG16L-containing phagophores, thereby leading to autophagosome production and energy generation via autophagic degradation of organelles and other intracellular components [55–57]. Examination for ULK1 phosphorylation by immunoblotting indeed revealed an enhanced phosphorylation of ULK1 at Ser555 by BRUCE depletion (Fig 4A, lane 3 vs lane 1) and quantified (Fig 4B), supporting that the increased autophagy induction by BRUCE depletion is mediated by the activation of ULK1.

As an upstream protein kinase to activate ULK1, AMPK is activated by phosphorylation at Thr172 both by its upstream kinases and binding of ADP and AMP (which inhibits Thr172 dephosphorylation and causes allosteric AMPK activation) [33–36]. Elevation in cellular AMP and ADP concentration, or reduction of ATP concentration are signals of a compromised cellular energy state [58–61]. To examine whether BRUCE depletion has resulted in AMPK activation, we examined the level of phospho-AMPK (Thr172) by immunoblot and this level was indeed higher in BRUCE depleted cells than that in controls (Fig 4A, lane 3 vs lane 1) which was confirmed by quantification of the immunoblotting results (Fig 4C). To confirm the specificity of shRNA in targeting BRUCE expression, another duplex siRNA (siBRUCE#2) also reproduced the same results of elevated autophagy flux, increased phosphorylation of ULK1 and AMPK (Fig 4D). To validate the effect to be BRUCE-specific, rescue experiments were performed in the U2OS-shBRUCE cell line that has been reconstituted with the expression of exogenous wildtype BRUCE (FLAG tagged) and the BRUCE depletion effect can be reversed (Fig 4E). Promoted by these compelling results, we examined whether the elevated active AMPK induced by BRUCE depletion is resulted from a lower cellular energy state. Measurement of the concentration of cellular AMP and ATP indeed showed a significant higher AMP/ATP ratio, i.e. lowered cellular energy in BRUCE depleted cells as compared to controls (Fig 4F). All together, these findings demonstrate a new “BRUCE-AMPK-ULK1 signaling axis” that indicates that BRUCE depletion regulates the induction of autophagy through activation of the energy sensor protein kinase AMPK and autophagy initiating kinase ULK1 (Fig 4G).

### Increase in autophagy co-occurs with enhancement of chemo-resistance in BRUCE depleted ovarian carcinoma cells

**Fig 5:**
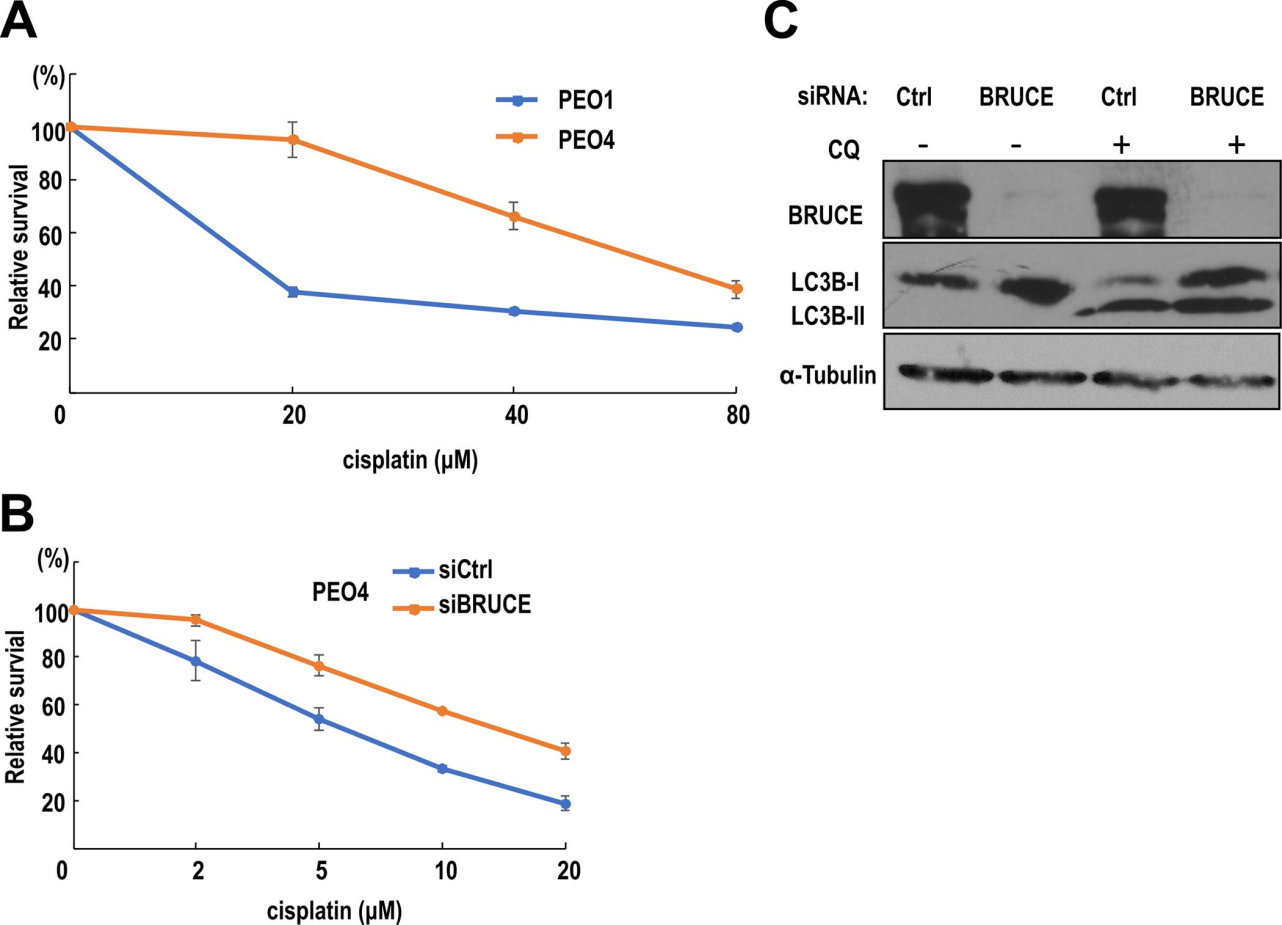
Increased autophagy co-occurs with enhanced cisplatin-resistance in PEO4 cells upon BRUCE knockdown. (A) PEO4 cells showed relative higher resistance to cisplatin treatment comparing with PEO1 cells by a cell survival assay. (B) BRUCE knockdown rendered PEO4 cells more resistant to cisplatin by a cell survival assay. (C) Immunoblotting showing autophagic flux in PEO4 cells was increased by BRUCE depletion.

Elevated autophagic activity can prolong cancer cell survival following chemotherapy and therefore enhance chemo-resistance in various types of cancers [62–64], including ovarian cancer [65]. Most ovarian cancer initially responds favorably to platinum-based therapy; however, recurrent disease often exhibits chemo-resistance resulting in poor prognoses. Therefore, understanding the cellular processes that contribute to chemo-resistance in ovarian cancer is critically important. It is known that high grade ovarian cancer cell lines PEO4 and PEO1 have differential response to platinum drugs, with PEO4 being more resistant than PEO1 cells [66, 67]. This differential response was confirmed in our lab by a cell survival assay with both cell lines exposed to cisplatin (Fig 5A).

To understand cisplatin resistance in PEO4 cells, and considering the likelihood of contribution of autophagy to chemo-resistance in ovarian carcinoma cells, we examined the impact of BRUCE depletion in PEO4 cells on chemo-resistance. Knockdown of BRUCE in PEO4 cells enhanced their cisplatin resistance (Fig 5B). Furthermore, similar to U2OS and MEFs, the autophagic flux in PEO4 cells was also increased by BRUCE depletion as shown by the accumulation of lipidated LC3B-II product following inhibition of the flux by chloroquine treatment (Fig 5C). This enhanced autophagy activity coupled with the co-occurring elevated cisplatin resistance in PEO4 cells suggest that enhanced autophagy is one of the mechanisms that contributes to cisplatin resistance in PEO4 cells.

## Discussion

BRUCE plays multiple critical roles in various cellular processes. The current study by analysis of multiple types of cell lines (human osteosarcoma U2OS cells, normal hepatocyte THL2, ovarian cancer cell PEO4, and murine MEFs) revealed a new function for BRUCE in the regulation of inherently connected processes of cellular energy and autophagy. We have identified a “BRUCE-AMPK-ULK1 signaling axis”, in which BRUCE depletion lowers cellular energy and in turn promotes the induction of autophagy through activation of the energy sensor protein kinase AMPK and the autophagy initiating kinase ULK1. The AMPK-ULK1-mediated activation of autophagy increases lysosomal enzyme functions and replenishes BRUCE deficient cells with energy from the degradation of intracellular contents. Based on these findings, we propose the following working model to explain how BRUCE regulates the cellular energy and autophagy (Fig 4G). Under normal growth conditions with sufficient nutrient supply, BRUCE expression is required for maintaining cellular energy at an optimal level. When BRUCE expression or function is lost, cellular energy level is reduced which activates AMPK to activate induction of autophagy for energy supplies. As a result, the ULK1-mediated initiation of autophagy increases, and the formation of autophagosomes and autolysosomes are increased, leading to enhanced autophagy coupled with elevated lysosome enzyme functions to meet the need of cargo degradation. This study provides insights for future investigation into the functional interplay between autophagy and other functions of BRUCE in the regulation of DNA damage response and apoptosis.

Starvation-induced autophagy signaling has been extensively studied. In contrast, the regulation of autophagy by cellular energy level is less clear. The current study provides insights into the function of autophagy in the survival of cells and tissues when BRUCE is lost or inactivated. One such example could be the contribution of elevated autophagy to chemo-resistance in ovarian cancer cells. Apparently, cancer cells can acquire chemo-resistance from elevated autophagy activity because elevated autophagy can provide a survival advantage through energy supplies from autophagic degradation of intracellular components/cargos. The elevated autophagic activity in BRUCE depleted PEO4 cells could explain enhanced platinum resistance. Our findings of BRUCE mediated regulation of high grade ovarian cancer cell autophagy provides an exciting therapeutic potential that leverages the broader impact of BRUCE on the regulation of autophagy in normal tissue homeostasis and disease.

Elevated autophagy activity can be a double-edged sword because the consequences of elevated autophagy are cell context dependent. When a normal cell needs to survive through a cellular energy crisis, downregulation of BRUCE could enhance autophagy to degrade intracellular components for sustaining cell viability and maintaining cellular function. In contrast, when cells with genome instability need to survive through a cellular energy crisis, loss of BRUCE could be detrimental to the body because the resultant higher autophagy activity could prolong the survival of these cells, leading to oncogenesis. It is known that cells deficient in BRUCE expression accumulate DNA damage and genome instability due to loss of its DNA repair function [18, 19], and become resistant to apoptotic cell death due to loss of its anti-apoptotic function [4, 6, 9, 10]. In this case, elevated autophagy combined with genome instability and escaped apoptotic cell death could make BRUCE deficient cells exhibit improved survival. Meanwhile the genome instability could contribute to tumorigenesis.

One critical question remaining is that of which physiological and pathological conditions cause BRUCE expression downregulation? BRUCE expression in cancers is altered in two ways, reduced or elevated (The Cancer Genome Atlas; TCGA data portal). In those types of cancers with low or absent expression of BRUCE, it would be interesting to assess whether tumor autophagy activity is increased and what impact this increase has on cancer therapeutic response, considering that elevated autophagy activity is often a contributor to enhanced chemoresistance [62]. Nonetheless, the “BRUCE-AMPK-ULK1” autophagy pathway provides new insights into how downregulation of BRUCE could impair cellular energy status and subsequently upregulates autophagy activity. Future studies are needed to identify the physiological impact of this pathway on tissue energy homeostasis and its implication in disease progression.

The impact of BRUCE depletion on the activation of autophagic activity demonstrated herein is consistent with a recent report that assessed BRUCE depletion’s role in blockage of starvation-induced autophagic activity [39]. The cross study consistency resides in the fact that each study addresses one of the two distinct cellular contexts. Our study addresses, in complete culture medium, how BRUCE depletion impacts autophagy, whereas the other study assesses starvation-induced autophagy. Our study was conducted in human cancer cells without any exogenous stresses such as starvation. In contrast, the other study was conducted with non-tumor cells (MEFs) under both BRUCE depletion and starvation conditions, with the goal of examining the function of BRUCE in starvation-induced autophagy. Therefore, cellular contexts and cell types are different, and the findings would be expected to be different. Our study demonstrates that BRUCE deficiency impairs cellular energy and activates autophagy to maintain energy homeostasis. The other study elucidates that BRUCE deficiency could block starvation induced-autophagy. These two studies considered together strongly suggest that BRUCE is a newly discovered regulator of autophagy, and its impact on autophagy is cell type and culture condition dependent. It is reasonable to speculate that at the beginning of a cellular energy crisis rendered by BRUCE deficiency, autophagic activity increases for cell survival; however, when the cellular energy crisis is prolonged, resulting in starvation, the autophagy program needs to be turned off, and cells are directed to the path of cell death. These theories require further validation in animals and humans as we believe that the complex and pleotropic functions of autophagy warrant that BRUCE promotes or inhibits autophagy in different tissues and organs.

How BRUCE normally regulates the homeostasis of cellular energy and how a low-energy state is generated by BRUCE depletion remains poorly understood. Nonetheless, BRUCE could regulate ubiquitin signaling by acting as both a scaffold and ubiquitin ligase to coordinate cellular energy sensing and metabolism. Given the importance of metabolic control of cellular energy by AMPK and control of autophagy by AMPK-ULK1, better understanding the regulation of AMPK-ULK1 pathway by BRUCE would provide potential therapeutic targets and new insights into how BRUCE determines the cellular energy state and cell fate in organs and tissues under physiological and pathological conditions.
